# Successful haploidentical stem cell transplantation with prophylactic administration of liposomal amphotericin B after invasive pulmonary zygomycosis

**DOI:** 10.1016/j.mmcr.2017.07.002

**Published:** 2017-07-04

**Authors:** Testuro Ochi, Yuta Katayama, Takeshi Okatani, Ryota Imanaka, Kohei Kyo, Mitsuhiro Itagaki, Shinya Katsutani, Koji Iwato, Hideki Asaoku

**Affiliations:** a Department of Hematology and Rheumatology, Tohoku University Hospital, 1-1, Seiryomachi, Aoba-ku Sendai-shi, Miyagi 980-0872, Japan; b Department of Hematology, Hiroshima Red Cross & Atomic-bomb Survivors’ Hospital, 1-9-6, Sendamachi, Naka-ku Hiroshima-shi, Hiroshima 730-0052, Japan

**Keywords:** Zygomycosis, Acute myeloid leukemia, Liposomal amphotericin B, Stem cell transplantation

## Abstract

A 54-year-old woman with acute myeloid leukemia (AML) achieved complete remission by induction chemotherapy, but developed zygomycosis after consolidation therapy. As zygomycosis could not be cured by liposomal amphotericin B and micafungin, left lower lobectomy was performed. As AML relapsed 7 months after onset, she received haploidentical stem cell transplantation under administration of liposomal amphotericin B. Despite experiencing severe acute graft-versus-host disease, she remains alive with no relapse of either zygomycosis or AML.

## 1 Introduction

The survival of patients with acute myeloid leukemia (AML) has improved with recent advances in chemotherapy and stem cell transplantation, mainly due to progress in infection prevention such as the introduction of granulocyte colony-stimulating agents, clean rooms, new antibiotics and antifungal agents [1]. In particular, antifungal agents such as itraconazole and voriconazole have played a major role by reducing mortality from invasive pulmonary aspergillosis. However, pulmonary mucormycosis remains a rare but life-threatening infectious disease associated with high rates of morbidity and mortality [2], [3].

Although pulmonary zygomycosis is often difficult to distinguish from invasive pulmonary aspergillosis, both of which are life-threatening fungal infections, it is important to distinguish them especially for hematologists, because voriconazole, the broad-spectrum antifungal agent recommended for the treatment of invasive pulmonary aspergillosis, has no activity against zygomycosis. In distinguishing these fungal infections, profound and prolonged neutropenia, and a history of diabetes mellitus, significant corticosteroid use, high-risk hematopoietic stem cell transplantation, and severe graft-versus-host disease (GvHD) favor the diagnosis of zygomycosis [4]. Voriconazole prophylaxis has also been associated with a diagnosis of zygomycosis [4].

As 10% of zygomycosis cases are reportedly diagnosed post-mortem or during the last 24 h before death [3], achieving a timely diagnosis of zygomycosis is very difficult. This difficulty might be caused by the lack of effective serum markers such as β-D-glucan and galactomannan antigen for invasive pulmonary aspergillosis. When zygomycosis is suspected, patients with hematological malignancies sometimes need to receive bronchoscopy before a definitive diagnosis of zygomycosis can be reached either histologically or by culture. However, bronchoscopy or transbronchial lung biopsy can be difficult to perform for patients receiving chemotherapy for hematological malignancy because of comorbidities such as thrombocytopenia or neutropenia. As mortality among patients with zygomycosis would increase day by day without amphotericin B-based therapy [5], we are often urged to start therapy before making a diagnosis of zygomycosis. As the “reversed halo sign” on chest computed tomography (CT) has recently been suggested as useful for early diagnosis of zygomycosis [6], CT is becoming helpful for earlier initiation of treatment.

Except in cases with relapse after a long remission, most AML patients in first relapse require allogeneic stem cell transplantation (allo-SCT) to achieve cure [7]. However, physicians are often slow to perform allo-SCT for patients with a past history of invasive fungal infection. Such patients are clearly at higher risk of transplantation-related mortality due to invasive fungal infection [8], but depriving them of the opportunity to receive allo-SCT often means losing the possibility of cure. As some patients can reportedly receive allo-SCT safely under full-dose antifungal therapy during the transplant period [9], we should not consider a history of invasive fungal infection as a definitive contraindication for allo-SCT. We describe the case of a patient who underwent haploidentical stem cell transplantation for AML relapse after developing zygomycosis during consolidation therapy, which had been treated by liposomal amphotericin B (L-AMB) and surgery, and remains alive without recurrence of zygomycosis.

## 2 Case

**Fig. 2:**
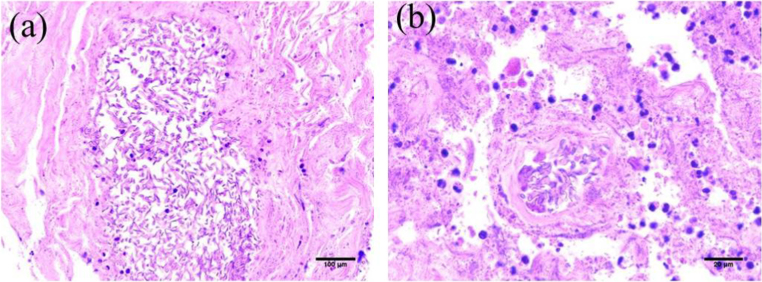
Pathological findings. (a) Hematoxylin and eosin staining, × 100, lung. Twisted or curved thick hyphae grow in various directions. (b) Hematoxylin and eosin staining, × 400, lung. Hyphae growing in a blood vessel are observed.

At Day 0, a 54-year-old woman visited our hospital with pancytopenia that had been identified incidentally in a health examination. Her medical history was significant for tonsillectomy due to tonsillitis at 30 years old and spinal canal stenosis that had been surgically treated at 52 years old. Family and psychosocial histories were unremarkable. She was diagnosed with AML classified as normal karyotype and Intermediate-II genetic group according to the criteria of European Leukemia Net [10]. After achieving complete remission by induction chemotherapy containing idarubicin, enocitabine, 6-mercaptopurine, and predonisolone [11], she received high-dose cytarabine therapy as post-remission therapy from day + 41. Voriconazole at 400 mg/day was used for fungal prophylaxis during induction and consolidation therapy. High-grade fever that did not respond to broad-spectrum antibiotics continued and acute chest pain occurred during the neutropenic phase. Because of persistent negativity of both β-D-glucan and galactomannan antigen and a focal round area of ground-glass attenuation surrounded by a ring of consolidation on CT of the chest (Fig. 1c), representing the so-called “reversed halo sign”, we started L-AMB at 600 mg/day and micafungin at 300 mg/day on day + 62 on suspicion of pulmonary zygomycosis. Although chest pain and fever improved with recovery of neutrophils, lung mass shadows decreased only slightly, despite intensive antifungal therapy (Fig. 1d). Left lower lobectomy was performed on day + 99 and zygomycosis was definitively diagnosed from the pathological specimen of lung (Fig. 2).

**Fig. l:**
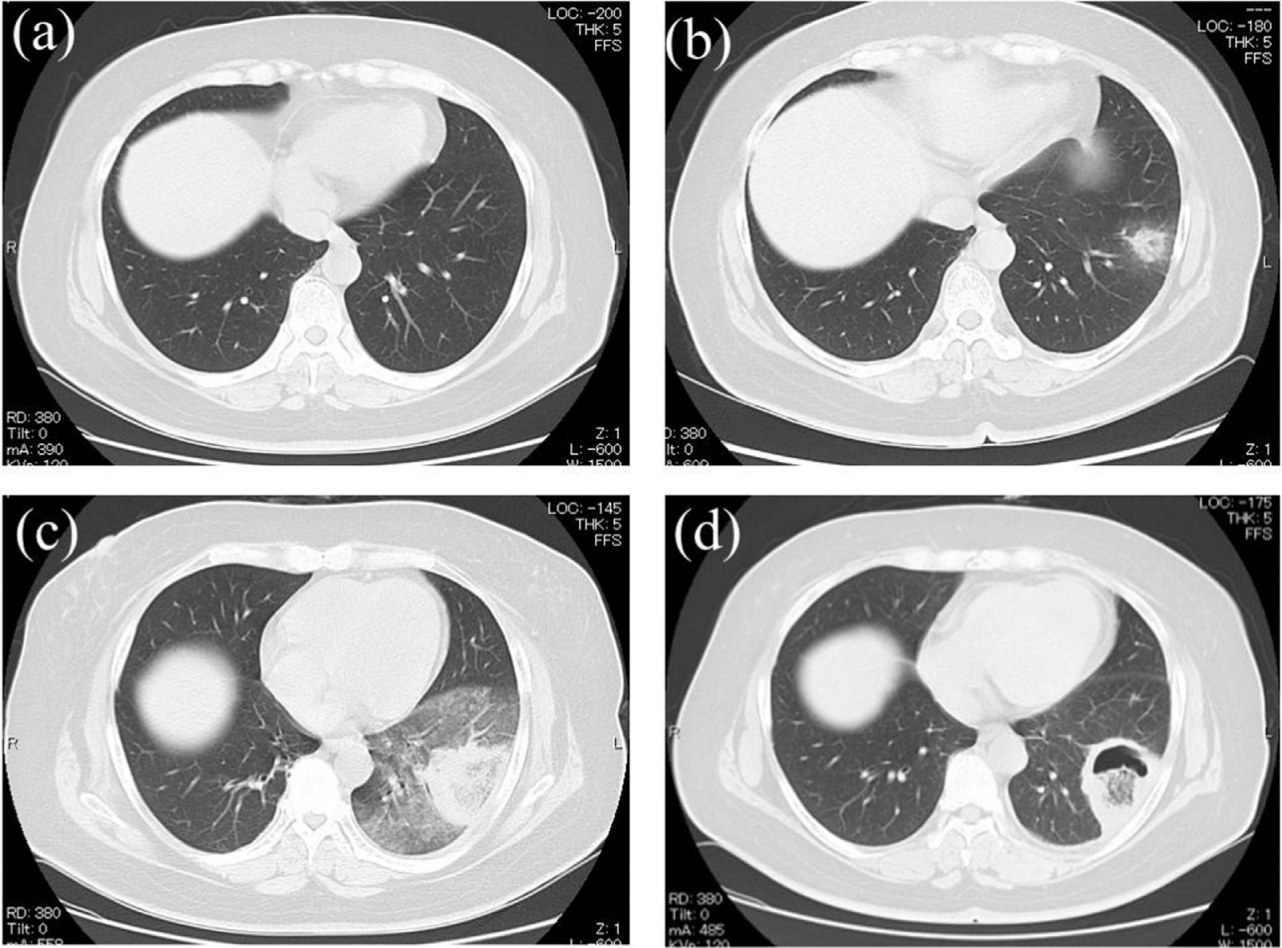
CT findings. (a) No abnormal shadow was observed at day + 47. (b) Halo sign observed at day + 54. (c) Reversed halo sign observed at day + 61. (d) Mass shadow with cavity observed at day + 89.

The patient received 2 cycles of enocitabine-based maintenance chemotherapy with administration of L-AMB at 300 mg/day as prophylaxis against pulmonary zygomycosis. As pancytopenia was prolonged after chemotherapy, bone marrow aspiration was performed on day + 208. On the basis of myeloblast proliferation (accounting for 13.5% of all nucleated cells) and trilineage dysplasia, we diagnosed AML relapse.

**Fig. 3:**
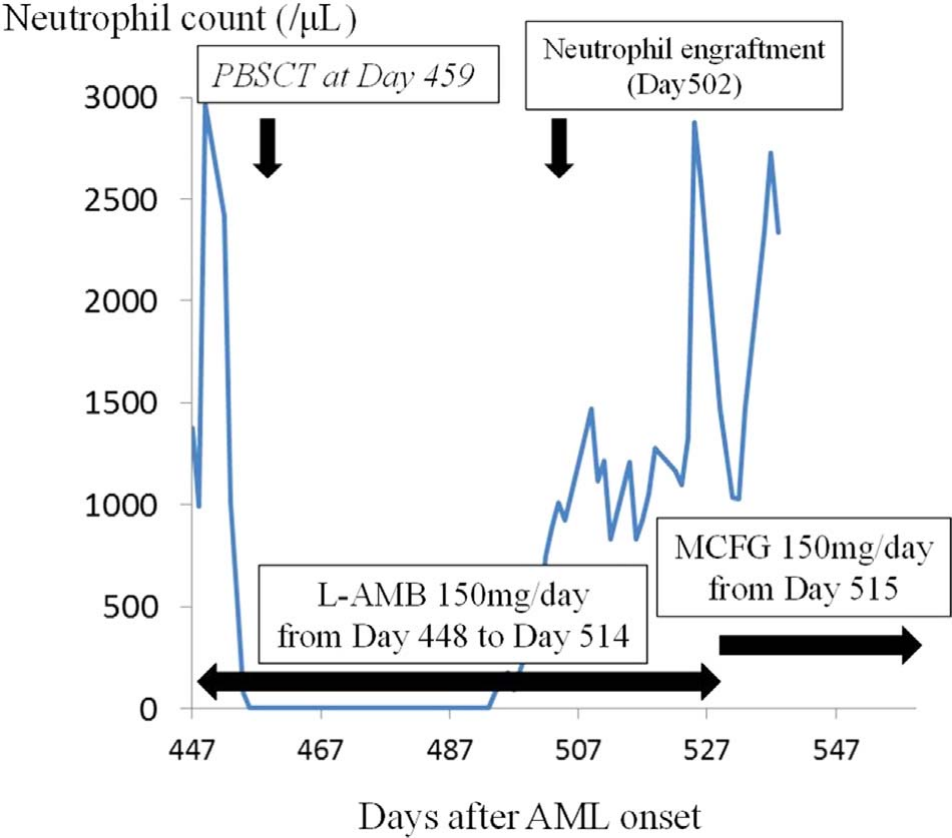
Clinical course before and after SCT.

While seeking a hematopoietic stem cell transplant donor, 7 cycles of azacitidine were administered. Although we gradually decreased the dose and finally stopped L-AMB on day + 189, no sign of mucormycosis relapse was identified. Unfortunately, as no human leukocyte antigen (HLA)-identical donor was located, the patient required peripheral blood stem cell transplantation from her HLA-haploidentical daughter on day + 459. She was conditioned with cytosine arabinoside, fludarabine, melphalan and total-body irradiation (2 Gy). Prophylactic treatment with rabbit anti-thymocyte globulin, methotrexate, tacrolimus and mycophenolate mofetil was administered to prevent GvHD. Rituximab was administered before stem cell transplantation to reduce levels of anti-HLA antibody. Macrophage colony-stimulating factor (M-CSF) (8 million units/day) was administered from day + 460 to + 469 (days 1–10 following SCT), then granulocyte colony-stimulating factor (G-CSF; 75 μg/day) was administered from day + 470 (day 11 following SCT). We started L-AMB at a dose of 150 mg/day for secondary prophylaxis against zygomycosis from day + 448 (day − 11 before SCT) (Fig. 3).

As neutrophils had not appeared by day + 480 (day 21 following SCT), bone marrow aspiration was performed. Bone marrow showed severe hypoplasia and engraftment analysis using multiplex polymerase chain reaction (PCR) for short tandem repeats (STR) revealed mixed chimerism, with recipient origin accounting for 79%. G-CSF was continued until neutrophil engraftment was achieved on day + 502 (day 49 following SCT).

Although stage 2 acute GvHD of the skin was identified on day + 502 (day 49 following SCT), no therapy was performed because recipient hematopoiesis was confirmed by previous engraftment analysis. However, not only did skin GvHD progress to stage 3, but also stage 3 acute GvHD of the gut arose, so methylprednisolone (mPSL) was initiated at 2 mg/kg/day on day + 548 (day 89 following SCT). As acute GvHD gradually improved, the dose of mPSL was tapered. On day + 514 (day 65 following SCT), dyspnea and high-grade fever arose during administration of L-AMB. These symptoms were relieved by stopping administration of L-AMB immediately and administering steroid. As this event was considered an anaphylactic reaction against L-AMB, no further L-AMB was administered beyond that day. Bone marrow engraftment analysis using multiplex PCR of STR on day + 566 (day 107 following SCT) revealed complete donor-type engraftment. The patient was discharged on day + 618 (day 159 following SCT) when mPSL was administered orally at a dose of 8 mg/body/day, but mPSL was continued at a dose of 8–16 mg/body/day because of chronic GvHD of the skin. She remains alive as of day + 918 (day 459 following SCT) without any relapse of zygomycosis or AML.

## 3 Discussion

As zygomycosis is a life-threatening infection because of its resistance to most antifungal agents and the difficulty of diagnosis, starting effective antifungal agents following early diagnosis of zygomycosis is crucial to reducing the risk of mortality [12]. The reversed halo sign, as a focal round area of ground-glass attenuation surrounded by areas of consolidation on chest CT, is seen more often in patients with zygomycosis than in those with invasive aspergillosis, and is considered very useful for early diagnosis of zygomycosis [6]. In this case, as the reversed halo sign appeared under voriconazole prophylaxis, which is a risk factor for mucormycosis [4], administration of liposomal amphotericin B was immediately initiated on suspicion of mucormycosis. Although the echinocandins do not exert activity against zygomycosis, we added micafungin on expectation of a synergistic effect [13].

Left lower lobectomy was performed following primary treatment with L-AMB in this case. In a retrospective analysis of 28 cases receiving primary treatment of zygomycosis with liposomal amphotericin B, nearly half of patients underwent surgical resection [14]. Surgery added to antifungal chemotherapy has also been reported to improve zygomycosis patients, although that study was not limited to patients with pulmonary zygomycosis [2]. If such treatment is not suitable for all zygomycosis patients, surgery might be considered in more limited circumstances, such as patients in whom zygomycosis lesions persist despite provision of appropriate antifungal treatments, such as L-AMB.

As few drugs have been confirmed as effective against zygomycosis despite the great recent progress in antifungal treatments, few options are currently available for zygomycosis prophylaxis. Secondary antifungal prophylaxis is recommended for as long as the patient is granulocytopenic or immunosuppressed by the Fourth European Conference on Infections in Leukemia guidelines [15]. Exactly which antifungal agents to use as secondary prophylaxis for zygomycosis then emerges as a major problem, particularly when survivors experiencing zygomycosis have to receive intensive therapies such as allo-SCT. Although posaconazole is a broad-spectrum triazole with antifungal activity against *Candida* spp., *Aspergillus* spp., *Fusarium* spp., and mucormycetes [16], a significant increase in the incidence of mucormycosis and fusariosis as breakthrough invasive fungal infections during posaconazole prophylaxis has been reported [17]. The effectiveness of L-AMB in the initial treatment of mucormycosis has been confirmed not only by experience [14], but also in a prospective study [18]. In fact, L-AMB as a first-line treatment was effective for zygomycosis. For that reason, L-AMB was selected for secondary prophylaxis against zygomycosis in this case. Fortunately, relapse of zygomycosis has not been observed in this case, but we must continue discussions of which antifungal agents represent suitable candidates for zygomycosis prophylaxis, and what doses are appropriate in the prophylactic application of L-AMB.

When using L-AMB for fungal prophylaxis before and after stem cell transplantation, major concerns should be held regarding severe renal dysfunction from combining the nephrotoxic L-AMB with nephrotoxic immunosuppressants such as tacrolimus or cyclosporine. As renal dysfunction leads to an elevated risk of GvHD caused by unstable concentrations of immunosuppressants, L-AMB seems likely to continue being regarded as difficult to use during SCT. However, as dose-dependent glomerular dysfunction associated with L-AMB was not found in a study of high-dose L-AMB [19], this drug appears safe to administer at lower doses.

In conclusion, we think that some room exists for carefully considering the use of L-AMB in secondary prophylaxis against zygomycosis for patients whose only option for cure of hematological malignancies with poor prognosis is intensive chemotherapy.

## Conflict of interest statement

The authors have no conflicts of interest to declare.

## Funding

This research did not receive any specific grant from funding agencies in the public, commercial, or not-for-profit sectors.

## Ethical form

Please note that this journal requires full disclosure of all sources of funding and potential conflicts of interest. The journal also requires a declaration that the author(s) have obtained written and signed consent to publish the case report from the patient or legal guardian(s).

The statements on funding, conflict of interest and consent need to be submitted via our Ethical Form that can be downloaded from the submission site www.ees.elsevier.com/mmcr. Please note that your manuscript will not be considered for publication until the signed Ethical Form has been received.
